# Children's Christmas Parties

**Published:** 1899-01-21

**Authors:** 


### The Hospital

A Journal of

The Medical Sciences and Hospital Administration.

Vol. XXV.—No. 643.] Edited by Sir Henry Burdett, K.C.B., and Solomon C. Smith, M.D., M.R.C.P. [Jan. 21, 1899.

### CHILDREN'S CHRISTMAS PARTIES.

The arrival of the doctor in the nursery as a natural sequel to the festivities of Christmas has for many generations furnished a theme to moralists, and an opportunity to those who would enlarge upon the fleeting character of all earthly enjoyments. The resulting discourses, in the days of our own childhood, were frequently made to turn rather upon the nastiness of the doses than upon the actual severity of the ailments for which they were administered; but although the progress of pharmacy has done a great deal to remove the former reproach from medicine, it has had no corresponding influence upon the results of dissipation. It may well be doubted, indeed, whether these were ever more severe than at the present day, when, in spite of the supposed advances of knowledge, and of the boasted civilisation of the close of the nineteenth century, the Christmas vacations of quite young children are constantly occupied by a succession of entertainments suited neither to their nerves, to their morals, nor to their digestions. Few spectacles can be less pleasing, to those who look beneath the surface at their true significance, than a children's party of the description which has now become fashionable and popular, a gathering together of small people in an over-heated and over-crowded room in fine dresses, which are only calculated to restrain movement and to foster vanity, for the purpose of partaking freely of the most unwholesome dainties that can be provided for them, and of being subjected to the undesirable excitement of exhibitions which the spectators are too young to appreciate, although not too young to lose natural sleep as a result of them. A little girl of from five to ten years old, dressed out in costly garments and adorned with jewellery, or a boy of the same age in satin and velvet, each deeply impressed by nursery admonitions on the importance of taking care of the fine clothes, which will have to be displayed again next week, is as far removed from the natural carelessness of childhood as an old man or an old woman; and a change so completely subversive of the ordinances of Nature is not to be effected harmlessly. Children ought to be so dressed as to give them the greatest possible bodily freedom, and never in such a way as to impose upon them burdensome responsibilities for the integrity of lace or for the cleanliness of white satin. To convert them into costume dolls is to deprive them of half the pleasure which ought to be the heritage of childhood; or, in some instances, to replace that pleasure by rivalries which, if they are ever harmless, can only become so in maturer years. A child who is puffed up by the consciousness of having better clothes than her playfellows is not likely to develop into an adult example of all the virtues.

Not the least remarkable consequence of the present style of children's entertainments is the way in which the juvenile appetite grows by what it feeds upon, as well as the way in which, with the customary frankness of tender years, its views upon the subject will be stated and enforced. We have heard of a child of six, at the close of a somewhat elaborate conjuring performance, inquire with precocious gravity, "Is that all?" and proceed to explain that at some other party, last week, they had animated photographs after the conjurer, and hot beef tea before they went away. "Are there only two presents each on the Christmas tree?" was another inquiry, elucidated by the further observation, "Mrs. Jones gave each of us three, and they were all much better than these." The entire business is essentially unwholesome; and is a sad falling off from the customs of the time when a children's party meant a good romp, a game at blind man's buff or hunt the slipper, a plain meal of simple food, and home to the sleep that comes of rational fatigue. Children cannot be manufactured into little men and women, and encouraged in the rivalries of men and women, without appreciable deterioration, not only of the simplicity of childhood, but of the physical health. We feel sure, not only that the doctor is as much in request in nurseries this winter as he ever was, but also that he is in request for more serious ailments. He has less to do with the casual too much cake which was the *fons et origo mali* of twenty years ago, and far more with conditions of nervous exhaustion, of a kind not always easily recovered from, and calculated, in no small number of instances, to exert a definitely prejudicial influence upon the growing frame. The cares of life, and the diseases which those cares entail, come soon enough to all of us; and nothing can be more short-sighted, or less wise, than to bring them into premature existence by treating our children as if they were little men and women. A children's ball is among the prettiest of sights; but the adults who look on with admiration would often be better employed if they would calculate the cost of the spectacle, not, indeed, in money, but in consequences.

